# Serological and molecular survey of *Leishmania* parasites in apparently healthy dogs in the West Bank, Palestine

**DOI:** 10.1186/1756-3305-5-183

**Published:** 2012-08-31

**Authors:** Omar Hamarsheh, Abedalmajeed Nasereddin, Safa Damaj, SamIr Sawalha, Hanan Al-Jawabreh, Kifaya Azmi, Ahmad Amro, Suheir Ereqat, Ziad Abdeen, Amer Al-Jawabreh

**Affiliations:** 1 Department of Biological Sciences, Faculty of Science and Technology, Al-Quds University, P.O. Box 51000, Jerusalem, Palestine; 2 Al-Quds Nutrition and Health Research Institute (ANAHRI), Al-Quds University, Jerusalem, Palestine; 3 Faculty of Pharmacy, Al-Quds University, Jerusalem, Palestine; 4 Leishmaniases Research Unit, Jericho, Palestine

**Keywords:** Serological and molecular diagnosis, *Leishmania*, Domestic dogs, West bank, Palestine

## Abstract

**Background:**

Canine visceral leishmaniasis (CVL) is caused by *Leishmania infantum* in all Mediterranean countries. The *Leishmania* parasite is transmitted by the bite of a corresponding sand fly vector and primarily maintained in nature by wild and domestic reservoirs, including dogs, foxes and jackals. Infected dogs are the primary reservoir host in endemic regions and are the most significant risk disposing humans to infection. The present study aimed at assessing the prevalence of infection with *Leishmania* and identification of *Leishmania infantum* in domestic dogs in the West Bank, Palestine.

**Methods:**

The infection rate among domestic dogs collected from seven districts in the Palestinian West Bank was investigated by examination of parasites in culture from the buffy coat using serological and molecular methods; based on ELISA, internal transcribed spacer 1 (ITS1) and cysteine protease (CPB) PCR.

**Results:**

Out of 215 dogs examined for *Leishmania*, 36 (16.7%) were positive in at least one method. Twenty three animals (11.5%) were positive for *Leishmania* DNA, whereas, ELISA and culture revealed 16 (7.5%), and 4 (1.5%) respectively. CPB-PCR on one of three culture-positive isolates revealed *Leishmania infantum* as the causative agent for *Leishmania* infection in dogs.

**Conclusions:**

Our study showed that canine *leishmania* infection is prevalent with varying degrees in all the seven studied districts in Palestine despite the absence of human VL cases in 4 of these districts. The causative agent was confirmed to be *Leishmania infantum*.

## Background

*Leishmania donovani* complex, which includes *L. infantum* (syn. *L. chagasi* in the New World) an obligate intracellular parasite, can cause systemic infection, which is fatal if not treated and considered an important zoonosis in Europe, Africa, Asia, and Latin America [1-5]. In the Mediterranean Basin, the causative agent of human and canine visceral leishmaniasis (VL) is *L. infantum*. This protozoan is primarily maintained in nature by wild and domestic reservoirs including dogs, foxes, and jackals [6-9]. Zonootic transmission of *L. infantum* in the Old World was described and well understood; in which females of phlebotomine sand flies of the subgenus *Larroussius* are the vectors of this zonoosis [10]. Infected dogs are the primary reservoir host in endemic regions, and are the most significant risk factor predisposing humans to infection [11]. However, other animals such as cats, red foxes and Jackals [12,13] were found accidentally infected.

Dogs have a wide range of clinical presentation due to infection with *L. infantum*, ranging from mild to fatal visceralizing disease. Host factors that determine clinical outcome are poorly understood and clinical symptoms include enlarged lymph nodes and hepato-splenomegaly due to parasitic invasion of the reticulo-endothelial system of phagocytic lymphocytes. The worldwide incidence of VL is estimated to be 500,000 cases/year, with more than 50,000 related deaths. Sudan, Indian subcontinent and Latin America were considered the most VL endemic areas in the world [14-16].

Detection of canine leishmaniasis by laboratory methods is crucial to prevent transmission of *Leishmania* parasites to humans, particularly as signs and symptoms of canine leishmaniasis are variable making clinical diagnosis a difficult task [8]. It has been demonstrated that both symptomatic (clinical symptoms are prominent) and asymptomatic dogs (*Leishmania* parasites diagnosed but clinically healthy) infected with the parasite are the sources of infection for humans transmitted by the bite of a sand fly [17,18]. Therefore, surveillance of canine leishmaniasis in endemic areas is very important to control VL in humans.

In Palestine, domestic dogs live in close proximity to humans along with other domestic animals which unfortunately when infected with CVL can pose a threat to human health, particularly in children. Reports from The Palestinian Ministry of Health (PMOH) indicated the existence of active VL foci in different areas especially in southern and northern parts of the country [19,20]. A study on domestic dogs from one Palestinian region in Jenin district in the north indicated high prevalence of VL among dogs [21]. However, no large scale survey that includes all Palestinian districts has been conducted since then. The need for such countrywide investigations in dogs may reveal their role as sources for subsequent parasite transmission to humans. Hence, information regarding prevalence and geographic distribution of canine infections are crucial for developing and monitoring strategic control measures by PMOH and health professionals in Palestine.

The purpose of this canine serological, parasitological and molecular survey was to: i) identify *Leishmania* infection in dogs, and thus their possible role as reservoirs, and ii) determine prevalence of *L. infantum* infection in domestic dogs in the West Bank, Palestine.

## Methods

### Study area

**Figure 1:**
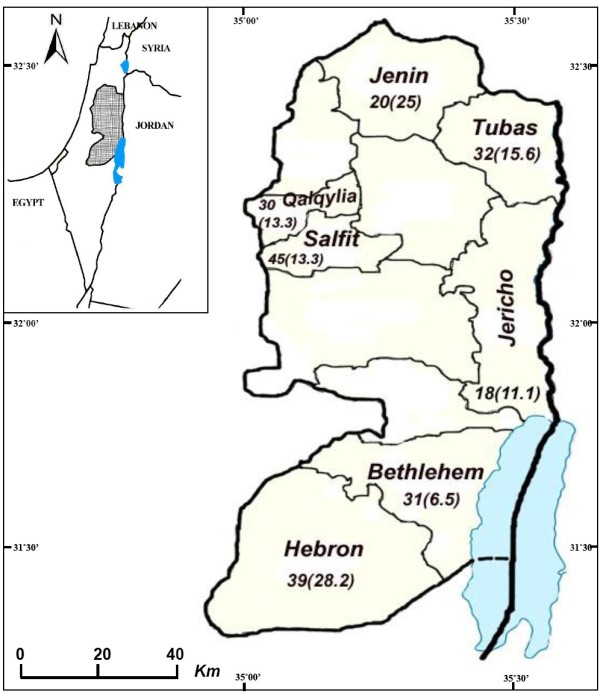
**Map of the West Bank, Palestine with study districts.** The numbers behind the name indicate the total number of dogs studied and the numbers in brackets are CVL prevalence in each district in (%).

The study was conducted in 14 villages distributed in 7 districts in the West Bank, Palestine; Jenin District (Silat Al-Hartheyeh and Al-Yamoun villages), Tubas (Sir and Tammoun villages) and Qalqilia (Azzoun village) in the north, Jericho District (Zbeidat and Marjna’jeh villages) and Salfit (Rafat, Kafr Al- Deik, and Zawieh villages) in the center, and Hebron District (Hatta and Beit Oula viallges) and Bethlehem (Obeidieh village) in the south (Figure 1,Table 1).The population of the studied areas is approximately 1,324,033 inhabitants according to the Palestinian Central Bureau of Statistics [22]. The study sites have different elevations; ranging from 260 m below sea level to 940 m above sea level (Figure 1). 

**Table 1:** **Distribution of* Leishmania *infected dogs by test method, district and locality**

**District**	**Locality**	***No*. dogs examined**	^**(a)**^**Detection of* Leishmania ***			^**(b)**^**Infected (%)**
			**Culture (%)**	**ITS1-PCR (%)**	**ELISA (%)**	
Jericho	Zbeidat	4			1/3 (33.3)	1 (25)
	Marj Na’jeh	6		1 (16.7)	1 (16.7)	1 (16.7)
	Marj al Ghazal	8				
Qalqylia	Azzoun	30		4 (13.3)		4 (13.3)
Tubas	Seir	2				4 (26.7)
	Tammoun	15		3 (20)	1 (6.7)	
Jenin	Silat Al-Hartheyeh	19		1 (5.3)	1 (5.3)	2 (10.5)
	Al-Yamoun	16		1 (6.3)	4 (25)	5 (31.3)
Salfit	Salfit	10			3 (33.3)	3 (33.3)
	Rafat	8		2 (25)		2 (25)
	Kafr Al-Diek	18		1(5.6)		1 (5.6)
	Zawieh	10				
Bethlehem	Obeidieh	31	1 (3.2)		1(3.2)	2 (6.4)
Hebron	Hatta	17	1(5.9)	8 (47)	3 (17.6)	8 (47)
	Beit Oula	21	1(4.8)	2 (9.5)	1(5.6)	3 (1.4)
Total		215	3/197 (1.5)	23/200 (11.5)	16/212 (7.5)	36 (16.7)

^(a)^ Positive samples/tested (%).

^(b)^ Infected: positive by ELISA or/and PCR or/and culture.

### Animals and sampling

Samples were collected from 215 domestic dogs (6 months or older) through door –to-door visits between January and June 2010. Demographical data about age, sex and type of breed of each dog was collected. Each dog was investigated for the clinical symptoms of leishmaniasis. This included the presence of skin lesions, nails deformation known as onychogryphosis, weight loss, hair loss (alopecia), lymphadenomegaly and weakness. Ten ml of peripheral blood (5 ml in each EDTA and plain tubes) were collected by cephalic venipuncture. Blood samples in plain tubes were allowed to clot. Samples were transported directly to the Leishmaniases Research Unit in Jericho. Plain tubes were centrifuged for 5 min at 3400 rpm, sera were separated in 1.5 ml labeled microcentrifuge tubes and stored in −80°C until use for determination of anti-leishmanial antibodies by ELISA. EDTA blood samples were immediately centrifuged for 10 min at 2500 rpm, the buffy coats containing leukocytes and presumed parasites were removed. About 50 μl were seeded in NNN culture media [23], while the rest of the buffy coats were used for DNA extraction.

### Enzyme-Linked Immuonsorbent Assay (ELISA)

*L. infantum* (MCAN/PS/2003/LQU-D1) promastigotes were harvested and used for antigen preparation as previously described [13]. Briefly, 96 well micro titer plates were coated with crude antigen and maintained overnight at 4–8°C. Wells were then washed and the sera were added in duplicate at dilution 1:100 then, incubated for 1 h at 37°C, washed three times with 0.1% Tween in phosphate buffered saline (PBS), pH 7.2. Protein (A) conjugated to horseradish peroxidase (Zymed Laboratories, Inc., San Francisco, CA) was added to each well as secondary antibody, and incubated for 40 min at 37°C. After further washes, chromogenic substrate (2,2-azino-di-3-ethylbenzthiazolihne sulfonate (ABTS); (Boerhinger Mannheim, Germany) was added, and the absorbance was read on an automatic Rosys anthos ELISA reader (Wals, Austria) at 405 nm. Negative dog sera (n = 23) from endemic and non-endemic areas were used to determine the cut-off value (0.3702), which was established as the mean absorbance value 3 times higher than the standard deviation (SD) from 23 sera from blood of uninfected dogs in endemic and non endemic areas. A sample was considered positive if its optical density (OD) was 3 times higher than the SD of the mean control group.

### DNA extraction and amplification conditions

DNA was extracted from buffy coat fractions using phenol-chloroform method [24]. Extracted DNA was purified using Nucleospin kit Extract II (Macherey-Nagel GmbH, Dueren, Germany) and DNA eluted in 30 μl TE (Tris-EDTA) buffer. Internal transcribed spacer 1 (ITS1) PCR was amplified using the primers LITSR ('5-CTG GAT CAT TTT CCG ATG-3') and L5.8S ('5-TGA TAC CAC TTATCG CAC TT-3') [25-27] amplification of *Leishmania* DNA was carried out in a 50 μl reaction volume, which contained 200 μM of each dNTP, 25 pmol of each primer, 1.25 μl DMSO, 1U *Taq* DNA polymerase (promega, Madison, WI,USA), 1x PCR buffer (10 mM Tris–HCl, pH 8.0; 50 mM KCl; 1.5 mM MgCl_2_) and 5 μl genomic DNA. The amplification conditions started with initial denaturation at 95°C for 2 min, followed by 35 cycles of denaturation at 95°C for 20 s, annealing at 53°C for 30 s and elongation at 72°C for 1 min and final elongation at 72°C for 6 min using thermal cycler (Biorad, C1000, USA). ITS1 PCR amplicons were analyzed on 1.2% agarose gels (FMC BioProducts, Rockland, ME) by electrophoresis at 100 V in 0.5 X TBE buffer (0.045 M Tris Borate and 1 mM EDTA). The DNA finally visualized by UV light after staining with ethidium bromide (0.5 μg/ml). A PCR result was considered positive when a 300–350 bp sized band was observed. Five ng positive and 2 μl distilled water for negative controls were added for each PCR run, this includes *L. turanica* and water, respectively. An inhibition control using 5 ng of *L. turanica* DNA was done for each sample to check for false negatives [28]. A dog was considered “infected” if at least one of these tests; ELISA, culture and ITS1-PCR, was positive.

Cysteine protease B (CPB) gene encoding for cysteine protease enzyme, was used to differentiate between *L. donovani* and *L. infantum*[29]. The CPB gene was amplified in a 25 μl-reaction ready-mix tube (Syntezza Bioscience, Jerusalem), 10 pmol of each primer; CPB forward primer: '5-GTTATTGGCTGCGTGGCTTG-3', and CPB reverse primer:'5- CGTGCACTCGGCCGTCTT-3', and 20 ng of mass cultivated leishmanial DNA. The amplification program started with initial denaturation at 95°C for 2 min, followed by 32 cycles of denaturation at 94°C for 30 s, annealing at 62°C for 1 min and elongation at 72°C for 1 min and final elongation at 72°C for 10 min. The PCR product was analyzed by agarose gel electrophoresis, followed by ethidium bromide staining and visualization under UV light as described previously. The test was considered positive for *L. donovani* when PCR product was 404 bp, and positive for *L. infantum* when PCR product was 365 bp.

### Statistical analysis

Chi-square test was used to determine the statistical significance. For all analyses, significance was indicated by a *P* < 0.05. Analysis was performed by SPSS (Version 12.0) statistical package.

### Ethical consideration

Study protocols and methodologies were revised and approved by the Ethical Committee at Al-Quds University. Written permission to perform the study was obtained from the PMOH. Prior meetings with community health authorities were held and the objectives were explained.

## Results

### Prevalence of CVL

Among 215 domestic dogs examined, the overall prevalence was 16.7% as reflected in the number of *Leishmania* positive subjects by at least one test (Table 1). A larger number of male animals, 168 (78%), compared to 46 females (21.5%) were sampled. Twenty nine (17.3%) males and 7 (15.2%) females were found to be positive.

Two out of 18 (11.1%) were found positive in Jericho, 4/30 (13.3%) in Qalqylia, 4/17 (26.7%) in Tubas, 7/35 (20%) in Jenin, 6/46 (13%) in Salfit, 2/31 (6.3%) in Bethlehem and 11/38 (28.9%) in Hebron (Figure 1, Table 1). The dogs examined fall into seven age groups; group 1 (≤1 year) consisted of 43 dogs (20%), group 2 (1–2 years) 52 (24.2%), group 3 (2–3 years) 17 (7.9%), group 4 (3–4 years) 15 (7%), group 5 (4–5 years) in 16 (7.4%), group 6 (> 5 years) in 13 (6%) and group 7 (unknown) in 59 (27.4%). The prevalence of *Leishmania* infection among each group ranged from 7% in age group 1 to 26.7% in age group 4 (Table 2). When dog breeds were examined; 113 dogs were of local breed, 12 Saluki, 16 of other types and 74 of unknown breeds (Table 2). Clinical examination of the dogs showed no clinical signs characteristic for VL; therefore all studied dogs were classified as healthy.

**Table 2:** **Distribution of* Leishmania *infection in dogs in the West Bank, Palestine by breed and age group**

**Biometric data**		**No. examined**	**No. positives**	**Prevalence (%)**	***P-value***
Breed	Local	113	20	17.7	1.000
	Saluki	12	2	16.7	0.117
	^a)^ Other	16	2	12.5	0.641
	Unknown	74	12	16.2	1.000
Age					
	≤ 1	43	3	7.0	1.000
	2	52	11	21.2	1.000
	3	17	2	11.8	0.758
	4	15	4	26.7	0.021
	5	16	4	25.0	0.050
	>5	13	3	23.1	0.027
	Unknown	59	9	15.3	1.0

^a)^ Other breeds include; Doberman (1), Boxer (2), Pointer (2), Rottweiler (1), Deporter (1), and Wolf (9).

### Isolation of Leishmania parasites

Isolation of *Leishmania* parasites was attempted from the buffy coat of 197 samples using NNN culture media. Three samples (1.5%) were successfully cultured. A WHO reference code was assigned to the one surviving isolate (MCAN/PS/2010/LRUJ-187), the other isolates were contaminated and eventually lost.

### Serological and molecular diagnosis

Two hundred and twelve out of 215 samples were analyzed by ELISA. Three blood samples showed signs of severe hemolysis and therefore were excluded. Anti-*Leishmania* antibodies were detected in 16 dogs (7.5%). Among the seropositives; 5 samples (14.3%) of 35 dogs collected from Jenin district, 4 samples (10.5%) of 38 dogs from Hebron district, 2 (11.1%) of 18 dogs from Jericho, 1 (6.6%) of 15 dogs from Tubas, 3 (6.5%) of 35 dogs from Salfit and 1 sample (3.2%) of 31 dogs from Bethlehem. However, all serum samples collected from Qalqylia district were negative (Table 1).

Out of 215 samples, 200 were analyzed by ITS1 PCR (15 were lost), *Leishmania* DNA was detected in 23 samples (11.5%). One dog showed positive result on all diagnostic methods used; culture, ELISA and PCR. On the other hand, three samples were positive by ELISA and PCR, simultaneously, while one sample was positive in ELISA and culture but not by PCR (Table 1). Twelve samples were ELISA positive but PCR negative, while, 19 were ELISA negative but PCR positive.

**Figure 2:**
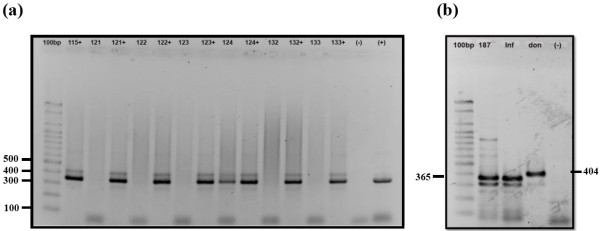
**Detection of* Leishmania *using agarose gel electrophoresis.** ITS1 and CPB-PCR products generated from amplification of DNA in buffy coat of dogs from the West Bank, Palestine. (**a**) ITS1-PCR produced a single band (310 bp) diagnostic for * Leishmania * DNA. 100 bp; DNA size marker, lanes from 115–133 represent dog samples, numbers with (+) represent inhibition control (* L. turanica * DNA added to sample). Lane 124 represent a positive sample, (−) represent negative control, and (+) represent positive control. (**b**) Identification of * Leishmania * isolated from a dog with CVL by CPB- PCR. 100 bp DNA size marker, 187; sample MCAN/PS/2010/LRUJ-187 isolated from asymptomatic dog. Inf; * L. infantum * (positive control), don; * L. donovani * (positive control) and (−) negative control.

The distribution of ITS1 positive samples showed that 10 (26.3%) positive samples were collected from Hebron district, followed by Qalqylia district with 4 (12.5%) positives and Tubas district with 3 (20%) positives. Since ITS1-PCR was not able to differentiate between *L. donovani* and *L. infantum*, cysteine protease B (CPB) PCR was used. MCAN/PS/2010/LRUJ-187 isolated from a healthy dog in Bethlehem showed a band size of 365 bp diagnostic for *L. infantum,* which is the causative agent of CVL in the Mediterranean region (Figure 2).

### Epidemiological implications

Factors such as age group, and breed were examined with prevalence of infected dogs. There was no significant correlation between animal gender and prevalence of *Leishmania* infection (*P* > 0.05). However, significantly higher prevalence was found among age groups 4, 5 and >5 year- old- groups (26.7%, 25% and 23.1% respectively with *P* < 0.05) (Table 2).

**Figure 3:**
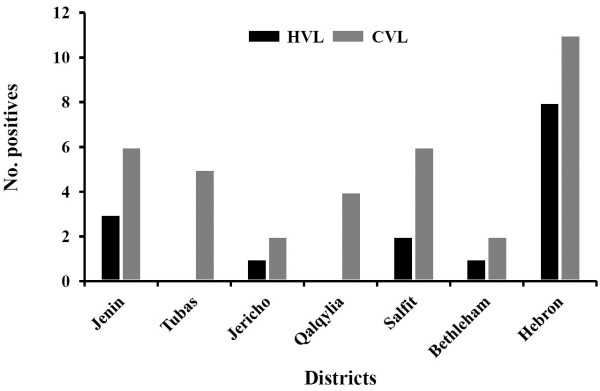
**Bar graph of Human Visceral Leishmaniasis (HVL) as reported by Palestinian Ministry of Health compared to study results of canine* Leishmania *infection in the seven Palestinian districts.**

When comparing prevalence of human VL (HVL) reported by PMOH from endemic areas in Palestine in 2010 and 2011 with *Leishmania* infected dogs in our study, consistency between both was noticed (Figure 3). The highest prevalence of VL was in Hebron district with 8 HVL and 11 *Leishmania* infected dogs, followed by Jenin with 3 HVL and 6 CVL, then Salfit with 2 HVL and 6 infected dogs. Jericho and Bethlehem showed only one HVL and 2 infected cases for each. However, Tubas and Qalqylia districts showed no HVL cases, but with 5 and 4 infected dogs, respectively.

## Discussion

This study indicated that *Leishmania* infection among domestic dogs is prevalent in the seven studied districts in Palestine. This comprehensive study, showed prevalence of *Leishmania* infection among domestic dogs (16.7%), higher than previously reported in Palestine [21] and neighboring countries [13,30], but lower than other Far East countries like China and Uzbekistan [31-33]. The domestic dogs surveyed in this study were asymptomatic. Those positive for canine infection are considered as hosts with a high capability of infecting sand fly vectors, thus forming a platform for the onset of human VL [34,35]. It is a common practice in Palestine to promptly cull dogs showing any clinical signs or symptoms for a certain disease. This is probably one reason of why none of the symptomatic dogs were included in the study. Furthermore, the long incubation period also contributes to the increased asymptomatic dogs at the expense of symptomatic ones.

The prevalence, depending on the age revealed that dogs aged > 1 year old had higher prevalence of *L. infantum* infection than those aged ≤ 1 year old. However, the difference was statistically significant among groups above 4 years (*P* < 0.05). It seems that there is a general tendency for older animals to have had more exposure to *L. infantum*. Older animals most likely have had more opportunities to come into contact with infective sand fly bites.

No significant differences in *Leishmania* infection were detected between female and male dogs, suggesting that gender of the host is not a crucial factor for CVL infection in Palestine. However, 78% of the tested dogs were males indicating sampling bias due to dog owner’s preference towards having a male dog.

Geographical distribution of CVL revealed that Hebron and Jenin districts are the most endemic areas in the Palestine, the high prevalence in Jenin district is in agreement with previous reports; 9.2% [21], and 5.3% [36]. However, a previous study by Amro *et al.* (2009) in Hebron district has shown an increasing number of human VL in many villages in this district and identified ownership of domestic dogs as a major risk factor of human VL. These results are consistent with our findings in this district. The dramatic increase of CVL in these districts is probably related to several environmental and ecological factors; expansion and urbanization of towns and villages, increased agricultural activity and livestock production. These changes have led to an increase in manure and waste production, providing good habitats for sand fly vectors to live and breed in as well as attracting stray dogs and wild canid reservoir hosts that may have contributed to the increase in VL incidence.

On the other hand, the absence of HVL in Qalqylia and Salfit districts in spite of the existence of *Leishmania* infection among dog populations could be described as the disease circulating in these two districts among dogs and for unknown reasons does not involve humans. This should be considered by the PMOH to prevent disease transmission from dogs to humans.

Only 3 out of 197 samples were positive by culture. The low number of *Leishmania* isolates from blood of asymptomatic dogs, was influenced by many factors; low parasite load in blood, and presence of parasites may temporarily fluctuate in peripheral blood, low inoculum size decreases secretion of the autocrine growth-regulating factor, thus slowing down parasite proliferation rate [37-39]. Two isolates were lost due to bacterial and fungal contamination and only one isolate (MCAN/PS/2010/LRUJ-187) was successfully maintained at the Leishmaniasis Research Unit in Jericho. This is due to difficult cultivation of *L. infantum* from blood and to the fact that blood samples from dogs are highly prone to contamination.

Comparing between the different diagnostic methods used, ITS1-PCR assay for CVL using DNA from buffy coat was shown to be more sensitive; samples were negative using ELISA and culture were found positive using PCR due to low sensitivity of culture and low level of antibodies in asymptomatic dogs.

Discrepancies between the diagnostic methods used were observed. Samples like no. 167 was positive by ELISA and culture but negative by PCR. Although PCR methods have high sensitivity, such negative results are probably due to the presence of *Leishmania* parasites in blood aliquots used in culture and missing in the aliquots used for DNA extraction; thus there is not enough to be amplified by PCR as the buffy coat is the third most convenient sample after spleen and bone marrow, but preferred in this study due to being less invasive, readily repeatable, more accepted by dog owners. Other explanations are based on the presence of PCR inhibitors. More sensitive molecular methods like kDNA, High-resolution melt analysis PCR (HRM PCR), Real-Time PCR, reverse dot blot assay could have increased PCR sensitivity.

The identification of *L. infantum* as the cause of CVL in the Bethlehem region is supported by molecular characterization using PCR-CPB of a *Leishmania* isolate from the buffy coat of a ten-year-old infected dog. The presence of dogs infected with CVL in the vicinity of Bethlehem complies with the zoonotic epidemiological pattern of CVL caused by *L. infantum* in the Mediterranean region, where canines are considered the main peridomestic reservoir of this parasite. Similarly, this is the case in other parts of the world like Central Asia and China [31,32].

The value of this work lies in two major points; it used the highest number, to date, of blood samples (215) collected from seven different districts in Palestine, and secondly the utilization of three different diagnostic methods; NNN culture, ELISA, and molecular diagnosis by PCR. This increased the accuracy of *Leishmania* detection (Table 1).

Limitations of this study included convenience sampling, which usually does not allow drawing of solid conclusions about the distribution of *Leishmania* infection among the study population. In addition most villages studied were chosen on the base of existence of HVL and accessibility. Thus the data do not have the power of a population-based random sampling study to provide a view of the real prevalence of *Leishmania* infection in the canine population. However, the data present a consistent picture of the *Leishmania* infection among dogs in Palestine.

Routine testing by The Palestinian Ministry of Health as well as Animal Service Units at The Palestinian Ministry of Agriculture is recommended as well as treatment of infected domestic dogs. Farm dogs in Palestine are less fortunate in receiving medical care by their owners, thus, exposing people in southern Palestine especially in Hebron district to the risk of being infected with HVL.

## Conclusions

The results of the present study revealed a high prevalence of *L. infantum* infection in domestic dogs in Palestine, especially in dogs above 5 years old, which has high public health significance due to its contribution to the transmission of infection to humans by sand fly vectors. Therefore, it is necessary to take integrated strategies, including efficient management measures to prevent and control *Leishmania* infection in domestic dogs, which will reduce HVL outbreaks.

## Competing interests

The authors declare that they have no competing interests.

## Authors’ contributions

All authors contributed significantly to this work. OH designed the study, participated in the field work and wrote the manuscript. AJ participated in the study design, carried out the field work and supervised the laboratory studies. SD did the laboratory studies, AN carried the molecular genetic studies. SW, AA, participated in the field work. SE revised the final version of the manuscript. ZA developed the idea of the study, managed the field studies and edited the final version of the manuscript. All authors read and approved the final version of the manuscript.
